# Actions of female sex workers who experience male condom failure during penetrative sexual encounters with clients in Cape Town: Implications for HIV prevention strategies

**DOI:** 10.4102/sajhivmed.v18i1.698

**Published:** 2017-04-04

**Authors:** Ferdinand C. Mukumbang

**Affiliations:** 1 School of Public Health, University of the Western Cape, South Africa; 2 Department of Public Health, Institute of Tropical Medicine, Belgium

## Abstract

**Background:**

Condom failure has always been found to coexist with condom usage, especially among sex workers.

**Objective:**

To describe the actions of female sex workers when they are faced with situations of condom failure.

**Methods:**

Using the survey design, the participants were selected through the snowball sampling method. Their responses were obtained using a structured questionnaire. A total of 100 questionnaires were analysed.

**Results:**

With respect to the immediate actions of sex workers after condom failure, 36% of the respondents continued with the sexual encounter after noticing that the condom was broken. Another 36% stopped immediately when they noticed that the condom had failed, but replaced the condom; 13% of the participants stopped the sexual encounter completely; 3% applied vaginal spermicidal foam; and 5% of the respondents stopped immediately and took a douche when they had the chance. For the actions within the next 24 hours of experiencing condom failure with a client, 53% of the participants did nothing; 4% sought counsel from a professional; 3% of the respondents took alcohol or drugs to forget the incident, 25% went to the clinic for assistance and 8% offered other responses.

**Conclusion:**

While continuing the sexual encounter without replacing the condom, taking alcohol and drugs or doing nothing could increase the risk of contracting HIV; however, actions like stopping the sexual encounter completely and visiting a clinic or a professional could make a difference between staying HIV negative or seroconverting. There is a need for targeted intervention to address issues of inappropriate behaviours after experiencing condom failure.

## Introduction

The global HIV prevalence among female sex workers is estimated at 11.8%, with the highest prevalence of 36% registered in sub-Saharan Africa.^1^ The rates of HIV among sex workers are relatively higher compared to the corresponding general population, making them disproportionately affected by the HIV pandemic.^2^ An Integrated Biological and Behavioural Survey of HIV prevalence and treatment rates among sex workers in South African cities conducted in 2015 revealed that an estimated 70% of sex workers in Johannesburg are HIV positive, while Durban’s sex workers have a 53% HIV infection rate and Cape Town’s almost 40%.^3^ It is, therefore, estimated that the HIV prevalence among sex workers is about 12 times greater than that among the general population.^4^ In South Africa, sex workers, their sexual partners and clients account for approximately 20% of all new HIV infections.^5^ Consequently, commercial sex is identified to play a significant role in the HIV epidemic in many developing countries, and it is progressively recognised as an essential part of HIV programming.^1^ Thus, they form part of the key target (most at risk) populations in the fight against the HIV pandemic globally.^6^

Working in the sex industry, biologically, structurally and behaviourally, predisposes female sex workers to acquire and thereby transmit HIV at a higher rate than the general population.^7^ As women are anatomically more predisposed to contracting HIV than their male counterparts, they are biologically more vulnerable. According to research, the delicate nature of the vaginal walls makes women, in general, three times more susceptible than men to sexually transmitted infections (STIs).^8^ As sex work is characterised by multiple sex partners and frequent coitus^9^ and also because sex workers sometimes indulge in ‘condomless’ sex with clients for more money,^10,11^ they are vulnerable from a behavioural perspective. Another important behavioural component that further exposes female sex workers is the fact that apart from indulging in commercial sexual activities, they also have non-commercial sex partners which in turn increase their exposure. As sex workers work in unsafe conditions and constantly encounter barriers to accessing condoms or negotiate condom use, they are at risk from a structural perspective.^2^ This is compounded by the ‘illegal’ status of sex work in South Africa. Physical and sexual violence from both clients and police have also been identified as conditions that propagate the transmission of HIV and/or AIDS in the sex industry as it compromises the ability of the sex workers to negotiate condom use. Because female sex workers are intimidated by their male clients, are terrified by violence and have limited access to women’s healthcare and prevention, they tend to practice inadequate HIV protection with their clients.^11^ Therefore, programmes designed to prevent the spread of the HIV infection in the sex industry should adopt strategies that address these components in a comprehensive manner.^12^

Sex worker–led prevention initiatives usually include sex education, training on STIs and HIV and/or AIDS, voluntary counselling and testing, and condom distribution and advocacy services. Most intervention packages contain at least three of the above-mentioned services and condom distribution forms an integral part of these packages.^4^ Therefore, condoms are considered an important component in the strategy to prevent the transmission of STIs, including HIV, in the sex industry as well as in general.^13^

### Trends

Effective condom use is recommended for people who engage in sex with multiple sexual partners^14^ such as sex workers; hence, condom use is highly recommended and encouraged within the sex industry. Although sex workers and their clients are exclusively responsible for their sexual health, working in the sex industry places the female sex workers at a higher risk of contracting HIV during commercial sex. The heightened risk that they face places more responsibility on the female sex workers to ensure that condoms are appropriately and effectively used.^11^

The effectiveness of condoms is based on the fact that they provide a more direct protection to both the sex workers and their clients. The emphasis on condom use is based on its practicability and its reported effectiveness in providing protection against STIs including HIV.^15^

Although male condoms are reported to be up to 98% efficient in preventing STIs including HIV with perfect use, condom failure has been identified to considerably interfere with the protective functionality, thus compromising its effectiveness. Common condom problems include breakage, slippage and leakage.^16^ Observational and prospective studies have estimated failure rates at 1%–13% during condom use.^17^ Condom failure is likely to occur in situations of poor condom fit; rough, lengthy or intense intercourse; diminished lubrication; or if the condom comes in contact with sharp edges.^18^ Higher rates of condom failure are identified among less-experienced users,^19,20^ anal sex and men having sex with men,^21^ situations of sex-perpetuated violence^22^ and among frequent users.^23^ The above-mentioned circumstances are common features of the sex industry. Consequently, condom failure is identified as an issue that should receive due consideration, especially among sex workers.

### Definition of terms

Sex worker: a person who indulges in sexual activities for a financial or material benefit, wherein sex is considered a revenue-generating activity rather than a deviant or criminal activity.^24^

Condom breakage: total breakage is defined as the number of condoms that reportedly open or split any time from opening the package to removing the condom from the penis, divided by the total number of condoms opened.

Condom slippage: partial or complete fall-off of a condom from a penis.

### Study objective

While many studies have explored issues around male condom failure among sex workers,^18,22,25^ an evidence gap was found with regard to what female sex workers do when they encounter circumstances of condom failure during penetrative sex with male clients. This study sought to describe the actions of female sex workers within the first 24 hours of experiencing male condom failure with a male client during penetrative sex.

### Contribution to field

These findings could inform national government departments and on-profit organisations (NGOs) working with sex workers such as Sex Worker Education and Advocacy Taskforce (SWEAT) and Sisonke to design interventions that address issues around condom failure. Their programmes should focus on empowering and equipping sex workers on what to do when faced with circumstances of condom failure and for possible lobbying for better and need-specific services such as healthcare facilities that can meet the needs in case of unanticipated exposure of sex workers to STIs including HIV.

## Methods

A cross-sectional, descriptive design was used for this exploratory pilot study. The study was designed as a small-scale version in preparation for a major study.^26^ The study catchment population included all the female sex workers who conduct sex work around the Cape Town Metropole. This includes both the roadside and the brothel-based female sex workers.

A sampling method known as the snowball sampling was employed.^27^ The first 10 participants included self-identified sex workers who are registered with SWEAT (seed participants). The seed participants were asked to recommend other self-identified sex workers willing to participate in the study. The investigator calculated the sample size using the estimate of 7500 for the Cape Town Metropole.^28^ Using the Raosoft^®^ sample size calculator, the investigator estimated the margin of error at 5%, confidence interval at 95% and the response distribution at 90%. Using these modalities, a minimum recommended sample size for the study was estimated at 136. Of the 142 participants recruited, only 100 questionnaires were completed correctly and admitted for analysis.

Although the snowballing method of data collection was employed to recruit the participants, the investigator ensured some degree of representativeness of the female sex workers’ population by making sure that participants were selected from the various areas renowned for sex trade. The investigator identified these sex work ‘hot spots’ from a sex worker population mapping that was done by SWEAT et al.^28^ The investigator recorded the regions the participants came from and using the sex work population mapping, identified those regions that were poorly represented. Although it is impossible to include a representative sample of all the communities sought,^29^ the investigator encouraged participants to recruit potential participants from the areas that had not been represented. The study participants were recruited during the months of December 2013 and January 2014 from the following sex trade ‘hot spots’: Bellville, Parow, Goodwood, Observatory, Brooklyn, Mitchell’s Plain and Khayelitsha. A R40 transportation fee was given to the participants who came in from the various areas to the SWEAT premises.

The investigator used self-administered questionnaires to collect the data in a standardised manner. The questionnaire used in this study comprised four sections. The first section explored the demographics of the study participants. The second section was related to substance use by the female sex workers. The third section dealt with their sex work experiences and the final section was based on condom use, condom failure (breakage and slippage) and actions of the sex workers after experiencing condom failure. This questionnaire was designed to gauge the actions of sex workers when they experienced condom breakage or slippage with their male clients. These questions explored the actions of the study participants immediately after they realised that the condom had failed as well as within the next 24 hours of the incident. This was based on the consideration that the first 72 hours are the most critical with regard to adopting actions that could mitigate seroconversion if a sex worker became infected as a result of condom failure.

To ensure the reliability of the questionnaire, simple and straightforward questions were used to reduce the chances of ambiguity and confusion. The questionnaire was adapted from an online commercial sex survey.^30^ The advantage of using instruments that have already been used by others is that they are recognised by the research community as effective measures in terms of conveying the meaning of the enquiry exactly as the research intended.^31^ Secondly, a copy of the questionnaire was verified by a research delegate of SWEAT for its appropriateness and completeness. Thirdly, the designed questionnaire was piloted among six participants who fitted the inclusion criteria. This pilot tested their comprehension of the questions to ensure that the questions were pitched at a level comprehensible to all the participants, including those who did not have any formal education. After the pilot study, corrections were made to the questionnaire to rephrase questions that were poorly understood by the pilot participants.

### 

#### Ethical considerations

This study received ethical approval from the Stellenbosch Ethical Research Council and permission from SWEAT. To guarantee a meaningful participation of the sex workers during the investigation, the study was conducted under the auspices of SWEAT. According to STELLA (a sex work–focused research institute),^32^ while conducting research involving sex workers, their participation under the ambit of a protecting body is important because it can prevent further stigmatisation and abuse of the sex workers.

The study participants were asked to sign a consent form confirming that their participation in the study is completely voluntary and that they understood the benefits and risks that were associated with their participation before responding to the questionnaires, which did not require any identification of the participants.

## Results

A total of 142 self-administered questionnaires were collected. Of the 142 questionnaires, 42 were incompletely filled and were thus discarded. The remaining 100 questionnaires were analysed using the IBM SPSS version 21. The results of the analyses are described under the following headings: demographics of the sample, condom use among the female sex workers, condom breakage and slippage, and actions of sex workers after condom failure. The actions of the sex workers are further divided into two parts: immediate actions and actions within the next 24 hours.

### Demographics of the sample

The demographics of the study participants are represented in terms of their age, marital status, educational background and race. Table 1 summarises the demographics of the study participants.

**TABLE 1:** Key demographics of study participants.

Variable	Representation	%
**Age distribution**		
Average	33.52	-
Range	19–60	-
Mode	30	-
**Age range distribution**		
10–19	1	-
20–29	42	-
30–39	35	-
40–49	15	-
50–59	7	-
60–69	1	-
**Educational levels**	**-**	
No formal education	-	18
Attended high school	-	58
Completed high school	-	8
Some college	-	5
College graduate	-	1
**Racial distribution**		
Black people	-	54
Mixed race people	-	41
White people	-	5
**Marital status**		
Single	-	58
Live-in	-	27
Currently married	-	8
Divorced	-	4
Widowed	-	1

### Condom use among female sex workers

**FIGURE 1:**
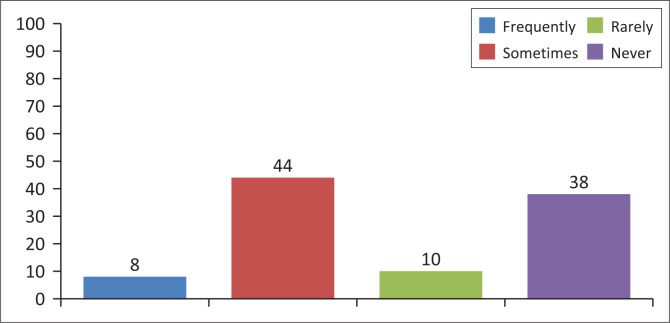
Responses of participants on condom usage.

To assess the rates of condom use, the investigator used the following parameters: frequently, sometimes, rarely and never. Almost all of the sex workers who participated in the survey (99%) indicated using condoms during their last sexual encounter with a male client during penetrative sexual intercourse (vaginal or anal). However, it is clear that consistent condom use is still an issue among sex workers working in the Cape Town Metropole. Almost half (44%) of the respondents attested to sometimes having sex without a condom for more money. Another 8% said that they frequently engaged in unprotected sex, while 10% indicated that they rarely engaged in unprotected sexual intercourse with their male clients. Therefore, 62% of the study participants at one point or the other of their sex work life had engaged in unprotected sexual encounters with their clients for more money. Nevertheless, 38% of the respondents reported that they had never engaged in unprotected sexual intercourse with their clients for more money. Figure 1 displays the results.

### Condom breakage and slippage

**FIGURE 2:**
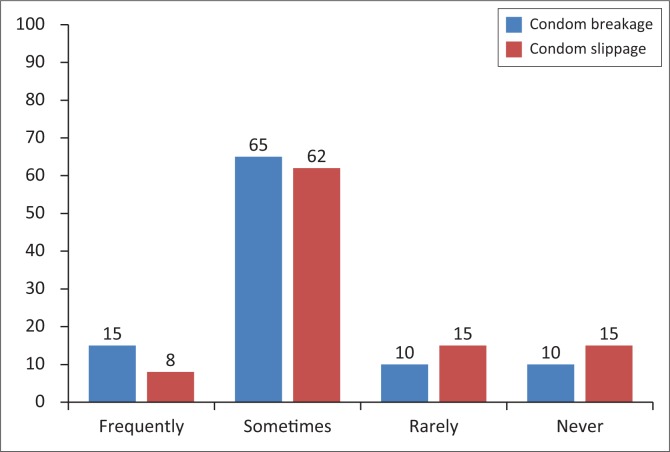
Responses of study participants on condom breakage and slippage.

Figure 2 represents the distribution of responses with regard to condom breakage and slippage. More people reported frequently experiencing condom breakage compared with condom slippage. The ‘sometimes’ responses of the participants are almost on par when asked how often they experienced condom failure or slippage. More people ‘rarely’ or ‘never’ had condom slippage compared to breakage. Figure 2 displays the responses of the participants on condom breakage.

Meanwhile, 90% of the participants confirmed having experienced condom breakage at some point or the other while working as sex workers, and 85% reported encountering situations of condom slippage. These findings mean that condom failure is a common occurrence in sex work among female sex workers.

### Actions of sex workers after condom failure

The first part focuses on the actions taken by the sex workers immediately after they noticed condom breakage or slippage. The second part focuses on their actions up until the 24th hour. The response percentages of the participants with regard to the immediate actions and actions within the next 24 hours of experiencing condom failure with a client are displayed in Table 2.

**TABLE 2:** Actions of female sex workers faced with condom failure during penetrative sexual encounters with clients in Cape Town.

Actions of sex workers	Response %	Mean	Std. Error
**Immediate actions**			
Continue to the end of the sex act	36	0.36	0.048
Stop immediately and put on a new condom	36	0.36	0.048
Stop the sexual encounter immediately	13	0.13	0.034
Apply vaginal spermicide foam	3	0.003	0.017
Stop and take a douche	5	0.005	0.022
Others	0	-	-
Non-response	7	-	-
**Within the next 24 hours**			
Nothing	53	0.53	0.049
Seek counselling from a professional	4	0.04	0.019
Take alcohol or drugs to forget the incident	3	0.03	0.017
Go to the clinic for assistance	25	0.25	0.043
Others	8	0.08	0.027
Non-response	7	-	-

#### Immediately after exposure

Regarding the actions of the sex workers immediately after they noticed that the condom was broken off or had slipped during the sexual encounter with a male client, the questionnaire offered six possible (options) actions (see Table 2). The participant responses showed that 36% of the respondents continued the sexual encounter with their clients to the end, even after noticing that the condom was broken or had slipped off. The same number stopped immediately after they noticed that the condom was broken or had slipped and put on a new condom, continuing the encounter to the client’s satisfaction. Around 13% of the participants told that they stopped the sexual encounter completely after noticing that the condom was broken or had slipped out during the sexual encounter. Three per cent revealed that they applied vaginal spermicidal foam immediately after experiencing condom breakage or slippage. About 5% of the respondents said that they stopped immediately and took a douche. None of the participants took any other action when faced with condom breakage and slippage. Finally, 7% did not offer any response because they had previously indicated that they had never experienced condom breakage or slippage while working as sex workers.

#### Within the next 24 hours

The second part of the action of sex workers was explored using the following question and response options: What do you do when you notice that the condom has slipped or broken when having sex with a client after the encounter is over until the next day? The response options are displayed in Table 2.

Slightly more than half (53%) of the participants reported doing nothing within the next 24 hours after noticing that the condom had failed. Meanwhile, 4% of the participants attested to seeking counsel from a professional after an experience of condom breakage or slippage, and 3% of the respondents revealed that they simply drank alcohol or took drugs to forget about the incident. A quarter of the participants (25%) indicated that they went to the clinic for help when they experienced condom breakage and slippage. The 8% who suggested other actions mentioned taking the morning-after pill for the prevention of pregnancy; however, they did not make a mention of anything they did to prevent the transmission of HIV. The last 7% offered no response as they had stated that they had never ever experienced condom breakage or slippage while having sex with a client.

## Discussion

### Condom use among female sex workers

The findings of this study revealed that many sex workers are making efforts towards using male latex condoms during penetrative sexual intercourse with their male clients. Nevertheless, most of these sex workers are ready to compromise their safety by engaging in unprotected sexual relations with their male clients for better pay rates. This fact is espoused by the finding that only 38% of the respondents reported never having unprotected sexual intercourse with their male clients for better pay. The other 62% of the respondents sometimes, rarely or frequently engaged in unprotected sexual intercourse for more money, representing the sex workers who use condoms inconsistently with their clients. These findings do not, however, tally with the findings of a study conducted by SWEAT et al.^28^ that explored the rates of condom use among sex workers in Cape Town. They observed that of the sex workers that engaged in vaginal sex with male clients, 64.3% reported using a condom at all sexual encounters with their clients. Notwithstanding, according to the same study, 45.6% of sex workers reported engaging in penetrative sex with a client without a condom. This figure is not far from the 38% of participants who admitted to indulging in unprotected sexual relations with male clients for better pay. Disparities of this nature are frequent because in most studies as in this one, condom use is self-reported and, therefore, potentially subject to bias.

Although there is a disparity between the exact percentage of sex workers who used condoms every time they had sex with a client, there is a substantial number of sex workers who are still engaging in unprotected sexual intercourse with their clients. Although other socio-cultural factors such as accessibility of condoms, client violence and forced unprotected sex, working under the influence of alcohol,^33^ substance use among sex workers^34^ and the influence of gatekeepers^35^ may contribute to the practice of unprotected sex, a factor that stands out is the need for financial stability.^36,37^ In essence, financial gain is the main reason why people employ themselves in the sex industry. Also, sex workers who are addicted to drug use are likely to engage in unprotected sexual intercourse for more money to pay for their habits, and so they need to secure more clients.^38^ Evidence accrued to date also links binge drinking, chronic alcohol use and other alcohol use disorders with unsafe sex and HIV transmission, and these disorders are common among sex workers.^39^ Alcohol and drug use are identified to compromise the thinking and decision-making capacities of the user. This explains why sex workers working under the influence of alcohol are likely to neglect using condoms during sex trade or perhaps do nothing when a condom breaks. Another important underlying factor that has been identified in the literature is power relations between the sex workers and their clients. Because the client pays for the sex act, they are perceived to hold more power in the condom-negotiation process, requiring negotiation skills on the part of the sex worker to tilt the power balance. If the sex worker is unsuccessful in the negotiation process, they will likely experience problems such as inability to resist the client’s pressure for unprotected sex.^40^

### Condom breakage and slippage

This study showed very high rates of condom breakage (90%) and slippage (85%) among sex workers in Cape Town. A study conducted in China revealed the prevalence of condom slippage and condom breakage during the three months before the survey was conducted at 36.2% and 34%, respectively.^22^ Although the rates of condom failure are different in both studies, there is a fundamental understanding that condom failure remains a prevalent problem against HIV preventive efforts in the sex industry. Although HIV prevention interventions have positively influenced the prevalence of condom use among sex workers in Cape Town, the risk of transmission of HIV among this population may remain relatively high as ‘unsafe’ sex stemming from condom failure remains prevalent. The situation is even more dire as mastery of condom use skills is always assumed among sex workers because of the understanding that they practice sex regularly. Unfortunately, condom failure always coexists with condom usage and condom use errors. The findings of this study revealed that condom breakage and slippage (condom failure) is fairly common among female sex workers in Cape Town. In a qualitative study conducted by Gurav et al.,^18^ sex workers identified rough sex in different forms – over-exuberance to violence – resulting from clients’ inebriation and use of sexual stimulants causing tumescence. They also identified excessive thrusting and sex that lasts longer than usual as the main causes of condom failure. According to a study conducted by SWEAT et al.,^28^ high levels of condom failure are associated with the Choice^®^ condom brand, the condom brand that is distributed by the South African government as a campaign against HIV transmission. While this study points to condom brands being a contributing factor in condom failure to an extent, studies have also shown that condom failure is more associated with condom use skills^21,41,43,44^ than the condom brand.

### Actions of sex workers after condom failure

It is recommended that if a condom breaks or slips off during sexual intercourse and before ejaculation, the parties involved should stop the sex act immediately and put on a new one before continuing.^16^ If ejaculation occurred, the receptive partner (female sex worker in this case) should squat and squeeze with vaginal muscles to remove excess semen, then wash their vulva, anus and surrounding areas with soap and water immediately, to reduce the risk of acquiring an STI including HIV.^45^ Inserting an applicator full of spermicide into the vagina as soon as possible is highly recommended. The spermicide in the applicator will help eliminate some of the organisms causing STIs. Females are warned against performing a douche because washing the inside of the vagina can alter the useful bacteria that protect the vagina from infection, and further push any sperm and bacteria into the cervix, thus increasing the chances of unwanted pregnancies and the transmission of HIV and STIs.^45^ After these required immediate actions, female sex workers are advised to visit a clinic or their doctor for further support.

#### Immediately after exposure

According to the survey, a third of the respondents continued their sexual encounter to the end, even after noticing that the condom had broken. This situation constitutes a very high-risk sexual activity, for both parties involved, as they are unaware of each other’s HIV status. Based on biomedical evidence and calculations, the risk of HIV infection is determined by the number of HIV-infected partners, the efficiency of HIV transmission and the number of unprotected sex acts with each HIV-infected partner.^5^ Inconsistent condom use, the length of working hours in the sex industry, a higher number of clients and high rates of condom failure are identified as proxy markers to the risk of HIV acquisition. Consequently, continuing a sexual encounter with a client after the condom has broken or slipped off is similar to having unprotected sex with the client. Therefore, the chances of contracting HIV, according to the biomedical formula, increases. Another third of the population reported that they stopped immediately after noticing that the condom had broken or slipped and that they put on a new condom to continue the sexual encounter to the end. Although this is better than the first option of continuing the sexual encounter in spite of the condom breaking or slipping, it still carries some elements of risk because of the prior mixing of the biological fluids.

Participants reported that they stopped the sexual encounter completely when they realised that the condom was broken or had slipped out during the sexual encounter. While this is identified as the best measure to prevent the spread of HIV and other STIs in the case of condom failure, it should be recognised that stopping the sexual encounter entirely at this stage is greatly influenced by the sex worker–client power dynamics. When a sex worker feels that they are in control of the situation, they could stop the sexual encounter completely and explain to the clients about the risks involved in continuing with the sexual activity. Conversely, if the sex worker feels less in control and intimidated by the client considering that she already has collected the clients’ money, the situation is left to the client’s discretion. This situation is potentially precarious because there is a high chance that the client will opt to continue the sexual encounter without any condom. This possibility is substantiated by a large number of studies portraying clients’ resistance to using condoms during the sexual transaction,^9,46^ supported by their willingness to pay more for unprotected sex.^5^ According to Gould and Fick,^47^ the demand for unprotected sex by clients creates most of the significant problems of sex workers. Gay et al.^48^ reported that most violence perpetrated on sex workers by their clients is based on their refusal to comply with the clients’ demands for unprotected sex, and up to a third of street-based sex workers have reported being raped by their clients. Therefore, violence or fear of violence has a role to play in determining what the sex workers do when faced with a situation of condom failure with a client.

A few of the participants revealed that they apply vaginal spermicidal foam after noticing that the condom had broken or had slipped off, while others said that they stopped immediately and took a douche. A survey, which was done among brothel and non-brothel-based sex workers, revealed that over 90% of sex workers washed their vagina with water mixed with Dettol^®^ or soda or turmeric after sex with their clients.^49^ This is a common practice among sex workers. Although, these practices could prevent sex workers from becoming pregnant, they might not be effective in preventing the transmission of the HIV virus and other STIs. On the contrary, taking a vaginal douche with hygienic substances eliminates the protective bacteria and alters the vaginal pH, thus exposing the vaginal lining to easy viral transmission. Martino and Vermund^50^ explained that the normal acidity of the vaginal environment can partly inactivate the HIV virus, which means that altering this pH level would expose the individual to various STIs including HIV. Consequently, the risk of contracting HIV after performing these acts remains higher, as the basis of these actions are at best myths.

Those who said they took other measures than those suggested in the questionnaire indicated that they took the morning-after pills. Some people believe that taking a birth control pill could prevent them from contracting HIV. According to Wilton, ‘the evidence, investigating the link between hormonal contraceptives and HIV transmission is inconsistent and limited’.^51^ Nevertheless, research shows that the progesterone contained in the birth control pills can increase the chances of contracting HIV in two ways. Firstly, it is suggested that progesterone can cause changes to the vaginal lining (decrease the thickness), which may increase a woman’s susceptibility to HIV infection. Research also shows that progesterone can increase the amount of virus (or viral load) in the vaginal fluid of women living with HIV, increasing their chances of transmitting HIV to others.^48^

#### Within the next 24 hours

The responses offered by the sex workers in this category represent their actions within the next 24 hours after a condom failure incident. The most popular response from the study participants indicated that the participants did nothing after the incident of condom failure with a client. Although it is unclear why they do nothing at all, a reasonable assumption is that they do not actually know what to do or where to go in situations of condom failure constituting high-risk exposure to HIV and other STIs. Although interventions to prevent HIV among sex workers have shown some success, they have had little impact on HIV transmission dynamics because they are implemented on very small scale, that most sex workers who need the prevention services do not have them available.^6^ In a study conducted by Parry et al.,^52^ they uncovered that many of the sex workers in Cape Town know about the treatment for HIV but they did not know where to go to get access to the antiretroviral medication. This is supported by Gay et al.^48^ who noted that most prevention interventions targeting sex workers focus on condom use and that just a few have advocated for sex workers to have equal access to antiretroviral medication. They also noted that sex workers have faced many of barriers to accessing healthcare. According to Scheibe et al.,^53^ discrimination, stigma and violence by police officers, clients, healthcare service providers, family members and community members have adverse impacts on the health and well-being of sex workers and thereby increases their vulnerability to HIV. Insensitive health workers might cause sex workers to avoid health facilities.^54^ Sex workers may have difficulties accessing both, post-exposure prophylaxis and legal services in situations of rape.^48^ These barriers could adversely impact on the health service–seeking behaviours of sex workers.

Three per cent of the respondents indicated that they indulged in the use of drugs (including alcohol, hormones and image- and performance-enhancing drugs) to forget about the incident that took place with the client. The association between alcohol use and sexual risks among female sex workers has been established.^55^ In addition, there is evidence that alcohol use by female sex workers and their clients can present problems for safer sexual and social interactions, which might lead to difficulty using condoms.^56^ Although the sex workers were aware of the dangers they faced with condom failure with a client, they indulged in drugs and alcohol to forget about the incident. Sex workers have reported using alcohol and other drugs to lower inhibitions and give them the courage they need to approach the clients.^52^ Nevertheless, as revealed by some of the sex workers in this study, those who had developed drug and alcohol dependency had the propensity to use these substances to drown their worries and escape from the reality of their problems. Thus, according to Chen et al.,^55^ alcohol use in commercial sex should be considered an occupational hazard that requires immediate intervention.

Notwithstanding, a fair number of sex workers reported having attended a clinic (27%) and making use of the services of a professional (4%) within 24 hours of experiencing condom failure. This makes a total of 31% of the participants who displayed positive actions within the 24 hours following an experience with condom failure. This behaviour is also reported by Richter, ^57^ who observed some positive attitudes of the sex workers towards healthcare services tailored to sex workers’ needs, such as sex worker consultation, peer education and empowerment initiatives. She explained that strategies to mitigate sex workers’ risk for HIV and ill health by ensuring access to proper and sensitive healthcare and education in Rustenburg were successful.

### Limitations on the study

The snowball sampling method limits the possibility of generalising the research finding to the general population identified because it has a high possibility of producing biased samples.^29^ This method had its downfall in that generally, participants tend to refer only those who might have similar preferences, dislikes and behavioural patterns, thus the sample may include an over-representation of individuals with numerous social connections who share similar characteristics.^58^ Another limitation of this study is related to the sample size. Some potential problems with studies done on sex workers using self-administered questionnaires are that the respondents are likely to be biased when responding to questions on topics such as condom use and drug habits.^59^ To minimise the chances of this, the participants were reminded of the importance of responding to the questions with honesty and the promise of anonymity throughout the study.

While the quantitative study could provide us with the spread of the problem, a qualitative study could provide us with participants’ perceptions and why they think and behave in a particular way with respect to the phenomenon in question.^60^

## Conclusion

Condom failure has always been found to coexist with condom usage and is often associated with condom use errors. This study explored what female sex workers do when a male condom fails during a sex act with a male client. Meanwhile, some of the actions such as continuing the sexual act without a new condom, taking alcohol and drugs or doing nothing at all could expose the sex workers more to contracting HIV; other actions such as stopping the sexual completely, paying a visit to a nearby clinic or visiting a professional could be the difference between staying HIV-negative or becoming HIV-positive. Unfortunately, many sex workers did nothing in the circumstance, avoiding seeking medical attention or specialised advice when they are faced with situations that increase their vulnerability to HIV. Although the National Sex Worker HIV Plan for 2016–2019 for South Africa provides for the reduction of risk behaviours and the creation of an environment for sex work that is safe for sex workers and clients including the provision of pre-exposure prophylaxis (PrEP) medication for sex workers, there is a need to understand the actions of sex workers when faced with circumstances that predispose them to contracting HIV for successful programming.
